# P04.84. A comparative survey study on integrative medicine in China and South Korea

**DOI:** 10.1186/1472-6882-12-S1-P354

**Published:** 2012-06-12

**Authors:** J Wang, H Lee

**Affiliations:** 1 San Francisco State University, Department of Health Education, San Francisco, USA; 2 College of Liberal Education, University of Keimyung, Keimyung, Korea, Democratic People's Republic of Korea

## Purpose

The modernization and professionalization of Traditional medicine has been achieved in both China and South Korea under different state and institutional policies and contexts. The objective of this survey study is to compare both Traditional (TM) and Western (WM) medicine doctors’ perspectives on the integration of Western and Traditional medicine in China and South Korea.

## Methods

The survey questionnaire was conducted during 2004-2006, once in South Korea and three times in China. The total survey subjects are 175 in China and 70 in South Korea. The survey focused on the following three aspects: (1) doctors’ experiences of integrative medicine; (2) perceived effects of integrative medicine from doctors’ perspectives; and (3) models of integrative medicine.

## Results

Integrative medicine practices are increasingly popular in both China and Korea. 81.6% of Chinese medicine doctors and 32% of Western medicine doctors report experience with integrative medicine in China. In contrast, while 74% of Traditional doctors report experience with integrative medicine in South Korea, WM doctors in South Korea reported none. Doctors in China gave a slightly higher evaluation on the effectiveness of integrative medicine. Finally, there is considerable disparity in opinions on the “model of integrative medicine.” WM doctors in Korea and China generally proposed a “Western medicine dominant and Traditional medicine complementary model.” In contrast, most Korean doctors proposed a “Korean medicine and Western medicine 1+1 model”, and most Chinese doctors believed in Integrative Medicine as a “third medical system” model.

## Conclusion

Despite the fact that most traditional doctors believe that there are theoretical and clinical conflicts between Traditional medicine and Western medicine, a large percentage of them think it’s possible to integrate. The variety of integrative medicine models in both countries reflect the intertwined power struggles of multiple medical systems that are continuously influenced by historical legacies and national health policies.

